# Study on Prevalence of lodine Deficiency Disorder and Salt Consumption Patterns in Jammu Region

**DOI:** 10.4103/0970-0218.39236

**Published:** 2008-01

**Authors:** Imtiyaz A Bhat, Iqbal M Pandit, S Mudassar

**Affiliations:** Department of Community Medicine, Sher-i-Kashmir of Medical Sciences, Soura, Srinagar, Jammu and Kashmir, India; 1 Department of Clinical Biochemistry, Sher-i-Kashmir of Medical Sciences, Soura, Srinagar, Jammu and Kashmir, India

**Keywords:** Crystalline salt, excretion, goiter, powdered salt, prevalence, urinary iodine

## Abstract

**Research Question:**

What is the situation of iodine deficiency disorder (IDD) and salt consumption in Jammu region?

**Hypothesis:**

The prevalence of IDD has decreased markedly as a result of medical as well as socio-economic factors.

**Objective:**

To assess the magnitude of IDD in Jammu region and also assess the salt consumption patterns in the region.

**Design:**

Cross-sectional study.

**Setting:**

Primary schools in both urban and rural areas.

**Study Tools:**

Clinical examination of study population for goiter, laboratory assessment of casual urine sample for urinary iodine estimation of I_2_ content of salt samples collected from sub-samples of study population.

**Participants:**

School children in the age group of 6-12 years were selected for study using WHO 30-cluster methodology, urine samples were collected from 15% of selected children and salt samples from 5% of sub-sample.

**Ethical Concern:**

No ethical issues were involved.

**Results:**

An overall goiter prevalence of 11.98% was observed in the region. Females had a prevalence of 16.1% and males 10.1%. The median urinary iodine excretion in the region was 96.5 μg/l (range: 29.0-190.0 μg/l). Forty-nine percent of subjects had biochemical iodine deficiency with 6.7% having moderate and 42.53% mild iodine deficiency. In Jammu region, 74.47% of households consume powdered salt with 98.17% powdered salt samples having an I_2_ content of greater than 15 ppm.

**Conclusion:**

Iodine deficiency remains a public health problem in the region, though the region seems to be in a state of nutritional transition from iodine deficiency to iodine sufficiency.

Iodine is an essential element for thyroid function, necessary for the normal growth, development and functioning of the brain and body.(1) While iodine deficiency is known as to cause endemic goiter, its most deleterious effect may be on the developing brain of the foetus, ranging from mild brain dysfunction to irreversible intellectual impairment. It is the single most common cause of preventable mental retardation and brain damage in the world today.

Iodine deficiency disorder (IDD) is known to have a significant public health (PH) problem all over the world. About 1.5 billion people worldwide live at risk of IDD of which more than 655 million people are already affected with IDD. In India, about 200 million people live at risk of IDD, whereas more than 71 million people are suffering from goiter and other IDDs.(2)

One of the recent goiter survey's (1994) in the Kashmir valley reported astonishing figures with goiter prevalence varying from 33.9 to 55.02%, whereas no such study has been conducted in Jammu region, also no effort was made on salt consumption in any of the previous studies. More than a decade has passed and consequent to alarming figures that received media publicity, measures like awareness programmes ensuring iodized salt availability were taken in Kashmir region, whereas no such additional activity was seen in Jammu region. Thus, a need was felt to find as to whether any change in prevalence of disease and salt consumption pattern has occurred. What is the present status of goiter in other regions of Jammu and Kashmir?

We studied the prevalence of goiter in school children in both Jammu and Kashmir regions of Jammu and Kashmir state. The present paper describes mainly data from Jammu region, which consists of six districts, i.e. Jammu, Udhampur, Kathua, Rajouri, Poonch and Doda. In the present study, the urinary iodine status of a sub-sample of children was studied by determining median urinary iodine. The salt consumption patterns were also studied to see as to which extent the goal of universal salt iodization has been achieved.

## Materials and Methods

School children in the age group of 6-12 years were studied. The sample size of 768 was estimated expecting a goiter prevalence of 40% and accepted error of 5% using the formula.

The study population was selected using the WHO recommended EPI 30-cluster methodology.(3) A multistage sampling procedure was adopted to select the clusters (schools) for the study. In the first stage, a frame of educational zones in each district was drawn. Using random numbers, five educational zones were selected from each six districts in the region. Thus, a total of 30 educational zones were selected. Finally, using population proportion to size method, 30 schools (clusters), one from each educational zone, were selected as final sampling unit. A total of 30 students (15 boys and 15 girls) from each school were studied after being selected by systematic random sampling.

The selected children were examined for thyroid enlargement, as recommended by ICMR bulletin 1986. The thyroid enlargement i.e., goiter was graded as,(4)

G0 - No enlargement

G1 - A palpable enlargement

G2 - A visible and palpable enlargement

On-spot casual urine samples were collected from 134 of selected subjects in the school. Urine samples were collected in the wide-mouthed 100-ml plastic bottle. Few drops of toluene were added; bottle closed with an inner lid and transported under cold condition to laboratory at Department of Biochemistry (SKIMS). The urine samples were stored under refrigeration and analyzed by ammonium persulphate method for urinary iodine and results expressed in μg/l.

The study subjects were asked about the type of salt consumed at home. Five percent of study subjects were asked to bring 50 g salt from home in polythene pouches provided for the purpose. The salt samples were analyzed for I_2_ content by titremetric analysis and results were expressed as I_2_ ppm.

Appropriate statistical tests were applied to compare different variables. Chi-square test was used to compare goiter prevalence among age and sex groups.

## Results

A total of 943 SAC were examined in the region; in that 113 had goiter giving prevalence of 11.90%. No student had grade two goiter. Table 1 shows prevalence of goiter across various districts in Jammu region.

**Table 1:** Prevalence of goiter across various districts in Jammu region

Name of district	No. of students examined	G0	G1	G2	Total (%)	Remarks
Jammu	173	167 (96.5)	6 (3.5)	0	3.5	Gtr. not a PH problem
Kathua	155	139 (89.7)	16 (10.3)	0	10.3	Gtr. PH problem
Rajouri	156	139 (89.2)	17 (10.8)	0	10.8	Gtr. PH problem
Poonch	163	141 (86.6)	22 (13.4)	0	13.4	Gtr. PH problem
Doda	146	115 (78.6)	31 (21.2)	0	21.2	Gtr. PH problem
Udhampur	150	129 (86.00)	21 (14.0)	0	14.0	Gtr. PH problem
Total	943	830 (881)	113 (11.9)	0	11.9	Gtr. PH problem

The prevalence of goiter varied from 3.5 to 21.2%, the lowest being in district Jammu and highest in district Doda. Except for main district Jammu, iodine deficiency is a problem of PH importance in all other districts of Jammu region. The prevalence of goiter was higher in females than in males (16.12% vs 10.10%) as shown in Table 2.

**Table 2:** Prevalence of goiter according to sex and age group

Gender*	No. of students	G0	G1	G2	%Gtr.
Male	475	427	48	0	10.1
Female	468	403	65	0	13.0
Age group (years)*					
6-8	375	335	40	0	10.6
9-12+	568	495	73	0	12.8

χ^2^, df = 1, difference not significant *P* < 0.05

The prevalence of goiter was higher in 9- to 12-year-old children than in younger children though difference was statistically insignificant [Table 2].

### Urine analysis

A total of 134 casual urine samples were analyzed by ammonium persulphate method for urinary iodine excretion (UIE). The median UIE was 96.5 μg/l. The median UI was lowest in district Doda (87.01 μg/l), whereas the highest was recorded in district Jammu (120 μg/l) [Table 3]. The median UI was higher in males (98 μg/l). When UI values were graded as recommended by WHO (Box I), percentage of urine samples with UI < 20 μg/l - 0.00%, percentage of urine samples with UI 20-49 μg/l - 6.7%, percentage of urine samples with UI 50-99 μg/l - 42.53%. Forty-nine percent of subjects in Jammu region had biochemical iodine deficiency, in which 6.7% have moderate and 42.5% mild I_2_ deficiency, respectively.

**Table 3:** UI in various districts in Jammu region

District	No. of students	Median (UI μg/l)
Jammu	23	120
Poonch	23	99
Rajouri	21	102
Udhampur	21	91
Kathua	21	98
Doda	25	87

### Salt analysis

A total of 99 salt samples collected from children were analyzed for I_2_ content by iodometric analysis. The overall mean I_2_ content of salt samples was 16.95 ± 7.07 (15.62-18.20 ppm). The mean I_2_ content of powdered salt was 20.9 (SD = 6.64) higher than crystalline salt 4.75 (SD = 2.4) and the difference was statistically significant (*P* < 0.0001).

Nearly 74.47% of households in Jammu region consume powdered salt, whereas 25.02% consume crystalline salt; 98.17% of powdered salt samples had mean I_2_ content of greater than 15 ppm, but only 3.87% crystalline salt samples had such an I_2_ level.

## Discussion

The present study based on clinical examination of 943 SAC from six districts in Jammu region indicated a goiter prevalence of 11.98%. The goiter prevalence reported by our study was consistent with recent studies from the sub-Himalayan belt. Toteja *et al.*, on behalf of ICMR, reported an overall goiter prevalence of 4.78% in 15 districts of 10 states.(5) Sahu *et al.* reported a goiter prevalence of 13.1% from Orissa.(6) Biswas *et al.* reported a goiter prevalence of 11.3% from West Bengal and Umesh Kapil *et al.* reported a goiter prevalence of 12.1% from Kangra district of Himachal Pradesh.(7) In our study, the prevalence in different districts was as follows: Jammu had a prevalence of 3.5%, Kathua 10.3%, Poonch 13.4%, Doda 21.2%, Rajouri 10.8% and Udhampur had a prevalence of 14%.

The goiter prevalence reported by our study is consistent with results of recent studies from the region.(8) There has been marked improvement in the goiter situation in the region. This could be due to increased awareness about benefits of iodized salt, wide availability of iodized salt, improved socio-economic condition of the population with better purchasing power; more than 75% of population in Jammu region consumes powdered salt with more than 98.5% of powdered salt having an iodine content of greater than 15 ppm.

In our study, we observed higher goiter prevalence (13.0%) in girls than in boys (10.1%). Survey conducted by GOI revealed a prevalence of 29.3% in boys and 47.2% in girls in district Pilibhit and a prevalence of 7.3% and 17.0% in boys and girls, respectively in district Puranpur.(9) Sahu *et al.*(6) showed a prevalence of 23.124% in girls and 17.3% in boys in Orissa.

While studying the relationship of goiter prevalence with age, we observed that goiter prevalence was insignificantly higher in older children: 12.8% in 9- to 12-year-old children as against 10.6% in 6- to 8-year-old children. Zargar *et al.* reported a similar pattern: a goiter prevalence of 30.2% in children less than 6-year old and 50.6% in children greater than 12-year old.(10)

We analyzed 134 casual urine samples for UIE by ammonium persulphate method from Jammu region. The median urinary iodine was 96.5 μg/l. It was lowest in district Doda (87.00 μg/l) and highest in district Jammu (120 μg/l). Studies elsewhere have also shown similar results. Nayal al-Sayed during a goiter prevalence study, obtained an overall median UI of 150 μg/l.(11) In the present study, we assessed salt consumption patterns in Jammu region and also analyzed salt SAmples collected from children for iodine content. We found that 74.47% households in Jammu region are consuming powdered salt and 25.53% consume crystalline salt. The overall mean iodine content of salt was 16.7 ppm with 29.9 ppm for powdered salt and 4.75 ppm for crystalline salt, respectively. Nearly 98.17% of powdered salt samples in Jammu region had an iodine content of 15 ppm, whereas only 3.87% crystalline salt samples had adequate iodine content. Kapil *et al*. reported from Delhi that 41% of families consume salt with adequate iodine.(12)

## Conclusion

The study has shown that the region is in a state of nutritional transition from iodine deficiency to iodine sufficiency, yet complacency should not be allowed to overtake the measure to sustain drive to eliminate the menace.
